# Methodology for the determination of human respiration rate by using Doppler radar and Empirical Modal Decomposition

**DOI:** 10.1038/s41598-022-12726-z

**Published:** 2022-05-23

**Authors:** Miguel Hernandez-Aguila, Jose-Luis Olvera-Cervantes, Aldo-Eleazar Perez-Ramos, Alonso Corona-Chavez

**Affiliations:** 1 Instituto Nacional de Astrofísica, Óptica y Electrónica, Puebla, Mexico; 2 CONACyT-CICESE-Monterrey, Apodaca, Mexico; 3 Department of Electronics Engineering, TecNM Campus Oaxaca – ITO, Oaxaca,, Mexico

**Keywords:** Engineering, Electrical and electronic engineering, Health care, Techniques and instrumentation

## Abstract

In this work, a methodology is presented for the determination of the respiration rate of a person under test (PUT), the detection of movements, as well as the elimination of the spurious effects produced by the movements of the PUT. The methodology is based on Empirical Modal Decomposition (EMD) applied to the phase signal obtained by means of a quadrature Doppler radar operating in S band. The EMD allows to automatically eliminate the continuos component (CC) which is present in the phase signal since one of the main characteristics of the modes generated by the EMD is that its mean is equal to zero. On the other hand, the first mode of the EMD is used for the detection of movements while the sum of the second and third modes are used for the elimination of the CC drift caused by the DC drift and the high frequency components produced by the movements of the PUT. The proposed methodology was successfully tested in a PUT at rest and performing movements of the head, arm and combination of head, arm, and torso. The average respiration rate measured was 20.78 breaths / min with a standard deviation of 2.53 breaths/min.

## Introduction

In the 70's, Doppler radar began to be used for measure respiration rate of people. Since then, numerous studies have been developed for medical applications. Doppler radar offers the possibility of monitoring respiration rate without the need to have contact with the patient, which turns out to be a great tool for all medical personnel since their exposure to sources of contagion is reduced^1^.

**Figure 1:**
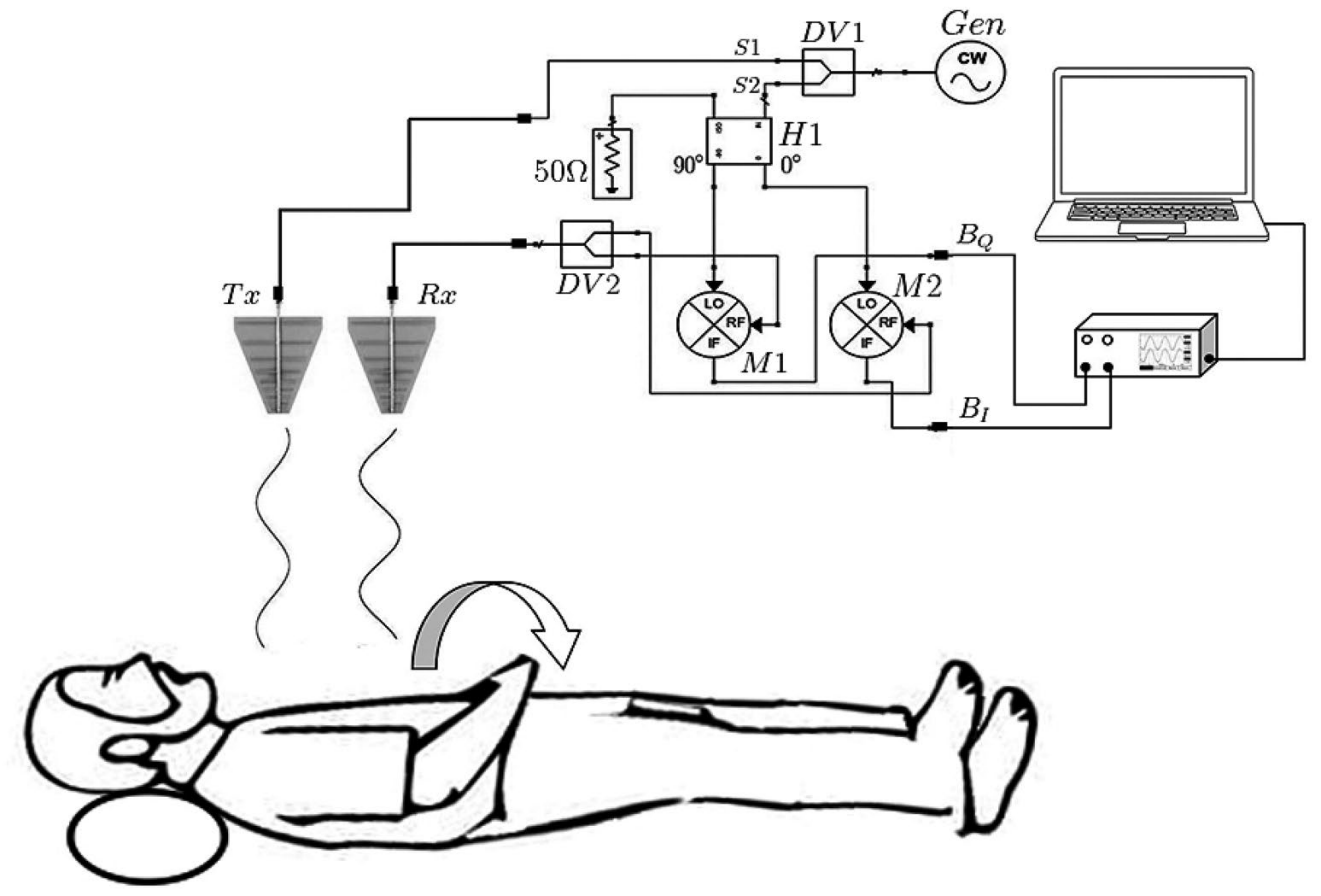
Experimental setup consisting of Quadrature Doppler radar, data acquisition system, and computer illustrating the relative location of antenna, and PUT.

A Doppler radar system generally consists of a transmitting antenna emitting an electromagnetic wave to the chest of the PUT which modulates the signal and reflects it towards the receiving antenna. The receiving antenna leads the modulated signal to a microwave mixer to convert the received signal to intermediate frequencies (IF) which are treated at the Digital Signal Processing (DSP) stage [see Fig. 1].

Relevant challenges of Doppler radars are the null point, the DC offset, the DC drift, the phase unwrapping, the problem of separating the heart signal from the breathing signal and the problem of breathing signal harmonics. The null point problem can be solved by using an RF phase shifter^2^. The DC offset can be treated by using a Doppler radar architecture fed with a stepped chirp signal^3^. Curve fitting can be used to separate the cardiac signal from the breathing signal^4^ as well as remove harmonic from the breathing signal^4^.

On the other hand, several studies have been proposed to remove the inevitable effects due to random movements. A proposed solution to eliminate Random Body Movements (RBM) is to use two Doppler radars in quadrature placed in front and behind of the PUT; this allows to obtain measurements in opposite directions of the random movement so that the movement can be compensated^5^. In another work it is proposed the use of a Doppler radar in quadrature together with a video camera to eliminate the RBM, so that random motion can be detected by the video camera and encoded as a phase signal that can be subtracted from the Doppler radar measurement^6^. Another proposed solution for the elimination of RBMs is the use of a curve fit to fit a polynomial function to the altered signal by an RBM, so that the fit function can be considered as the alteration and thus can be subtracted from the main signal^7^. It has also been proposed the use of a cyclostationarity analysis to extract the respiration rate and heart rate of a PUT, of signals obtained with a Doppler radar in quadrature and disturbed with noise and RBM, through the extraction of cyclostationarity moments of order 1 and 2 and the second cyclic cumulant^8^. On the other hand, the use of Empirical Modal Decomposition (EMD) has been proposed for the elimination of disturbances generated by small movements in the antenna of a quadrature Doppler radar. In (ref^9^) the authors are focused on the elimination of small random movements due to artifacts (the radar antenna) affecting the heart rate. It is important to mention that the problem of DC drift is omitted in (ref^9^) because the antenna movements are so small that they do not add a significant DC drift. Another proposed solution for the elimination of random radar movements is based on using an FMCW radar in quadrature to obtain the respiration signal of a PUT altered by random radar movements and use the autocorrelation operation to eliminate the random components of the signal and get an accurate measurement of the respiration rate^10^.

On the other hand, the DC drift issue consists of low-frequency (LF) components added to signals measured at Doppler radar. These LF components are mainly caused by both RBM and the temperature variations of radar electronics circuits. It is important to mention that the DC drift must be eliminated because it can generate errors in the breathing rate measurements. The DC drift can be removed by using a DC correction algorithm, which divides the signal with DC drift into sections and removes DC present in each of them^11^, polynomial regression^12^, detrending process^13^.

In this work, a quadrature Doppler Radar is implemented to measure the respiration rate of people. Empirical Modal Decomposition is an adaptive technique for the analysis of non-linear and non-stationary processes, which makes it ideal for the detection of disturbances generated by random movements, due to its non-linear and non-stationary nature. In this work, a methodology is proposed for the detection of movements of the PUT and for the mitigation of their effects in determining the respiration rate.

The proposed methodology was successfully verified in experiment for the determination of the respiration rate of a PUT, the detection of movements, as well as the elimination of the spurious effects produced by the movements of the PUT. The methodology is based on EMD applied to the phase signal obtained by means of a quadrature Doppler radar operating in S band. The EMD allows to automatically eliminate the CC which is present in the phase signal since one of the main characteristics of the modes generated by the EMD is that its mean is equal to zero. On the other hand, the first mode of the EMD is used for the detection of movements while the sum of the second and third modes are used for the elimination of the CC drift and the high frequency components produced by the movements of the PUT. The proposed methodology was successfully tested in a PUT at rest and performing movements of the head, arm and combination of head, arm and torso. The average respiration rate measured was 20.78 breaths / min with a standard deviation of 2.53 breaths / min. These values ​​were compared with a reference value of 19 breaths / min obtained through the technique of measuring the number of chest lifts of the volunteer for 1 min. It is worth mentioning that all respiration rate values ​​fall within the respiration range of a healthy adult, which is 15 to 20 breaths / min with a range of 24 to 28 breaths / min^14^.

## Implementation of an S-band Doppler radar

The diagram of the S-band quadrature Doppler radar used in this work is shown in Fig. 1. From this Figure, Tx and Rx are the transmitting and receiving antenna, respectively; Gen is a continuous wave generator, DV1 and DV2 are Wilkinson power dividers, H1 and H2 are hybrid quadrature couplers, and M1 and M2 are microwave mixers. The radar operation is as follows: the microwave signal (generated by Gen) is divided by DV1 into two signals of equal amplitude and phase. The resulting signal S1 goes to the transmitting antenna (Tx) to radiate the PUT’s chest. The signal bounces off his chest and, receiving antenna captures its reflections modulated by the PUT breathing. The received signal in Rx is divided into two signals of equal amplitude and phase using the DV2 divider. An H1 hybrid coupler separates the resulting signal S2 coming out of DV1 into two signals with equal amplitude and a 90 degrees phase difference. On the heterodyne stage, the emerging signals of the hybrid coupler and the emerging DV2 signals are mixed, obtaining the IF signals, harmonics, etc. Afterwards, the IF signals are digitized by an Analog to Digital Converter (ADC) contained on the Keysight U2702A card, a sampling rate of 25 Hz was used. Finally, the data is sent to the PC and processed in MATLAB ®.

The Ettus USRP B200 was used as a continuous wave generator Gen to generate frequencies from 70 MHz to 6 GHz. A pair of ZFSC-2-10G model Mini-Circuit’s power splitters that operate from 2 to 10 GHz were used as DV1 and DV2. The AMP M/A -COM 96,341 hybrid coupler operating in the frequency range from 2 to 18 GHz was used to obtain quadrature signals in H1. Two logarithmic antennas WA5VJB operating from 850 MHz to 6.5 GHz with a typical gain of 6 dBi, were used for the transmission and reception of the microwave signal.

The ZEM-4300 model Mini Circuits’s mixers operating with RF signals from 300 MHz to 4.3 GHz were used to obtain in phase ($B_{I}$) and quadrature ($B_{Q}$) IF signals. Both signals can be modeled by Eq. (1) and Eq. (2)

1 $$B_{I} \left( t \right) = DC_{I} + A_{R} \cos \left( {\frac{{4\pi d_{0} }}{\lambda } + \frac{4\pi x\left( t \right)}{\lambda } + {\Delta }\varphi \left( t \right)} \right)$$

2 $$B_{Q} \left( t \right) = DC_{Q} + A_{R} \sin \left( {\frac{{4\pi d_{0} }}{\lambda } + \frac{4\pi x\left( t \right)}{\lambda } + {\Delta }\varphi \left( t \right)} \right)$$

where *x(t)* is the function of periodic object movement, *d*_*0*_ is the nominal distance the object, *λ* is the wavelength of the signal transmitted by the radar, $\Delta \varphi$(t) is the phase noise, *t* is the time, *A*_*R*_ is the amplitude each IF signal and *DC*_*I*_ and *DC*_*Q*_ are amounts of DC offset caused by self-mixing of the mixers and by the reflection of static objects that is around the radar^15^.

From Eqs. (1) and (2), the Arctangent Demodulation, given in Eq. (3), can be obtained, which allows to measure the breathing and RBM elimination by digital processing of ${\Phi }\left( {\text{t}} \right)$. It is important to mention that the *DC*_*I*_ and *DC*_*Q*_ components must be calculated before applying the arctan function; this procedure is shown in the next section.

3 $${\Phi }\left( t \right) = \arctan \left( {\frac{{B_{Q} - DC_{Q} }}{{B_{I} - DC_{I} }}} \right) = \frac{{4\pi d_{0} }}{\lambda } + \frac{4\pi x\left( t \right)}{\lambda } + {\Delta }\varphi \left( t \right)$$

Finally, Fig. 1 shows the experimental set up in this study, which involves the detection of RBM and the elimination of its effects in the measurement of the respiration rate of a healthy male of 28 years, 80 kg of weight and a height of 1.78 m, lying down at a distance of 29 cm from the radar. The RBMs studied in this work are head, arm and torso movements.

## Proposed methodologies

### Proposed methodology to obtain the person’s breathing rate from signals affected by RBM

**Figure 2:**
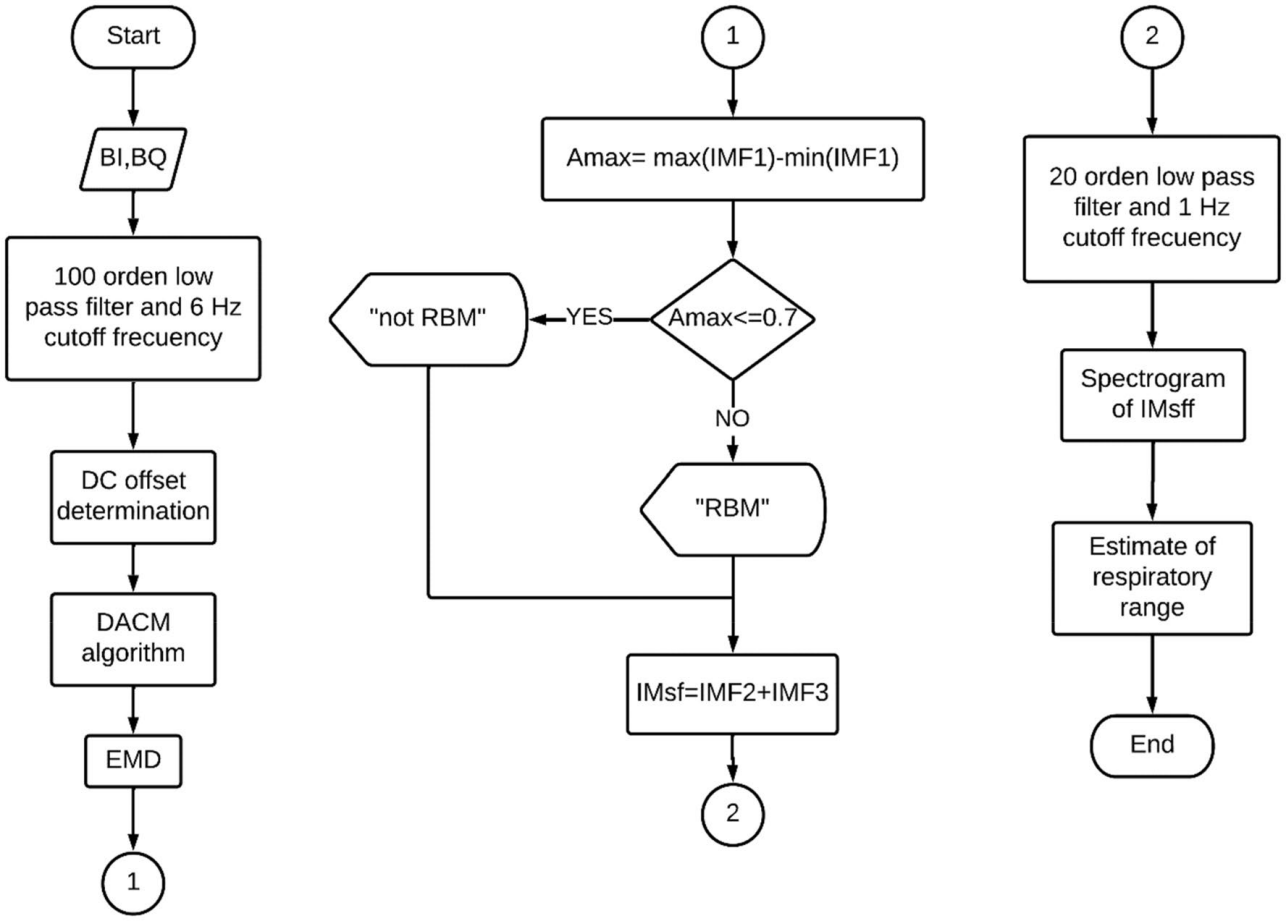
Flowchart of the proposed methodologies for RBM detection and for mitigating their harmful effects on the measurement of breathing rate.

Figure 2 shows the proposed methodology for RBM detection. In the beginning $B_{I}$ and $B_{Q}$ are measured by the ADC. The second stage involves the use of a 100-order low pass filter and 6 Hz cutoff frequency to eliminate high-frequency noise of B_I_ and B_Q_ without attenuating disturbances generated by RBM.

The third stage is to eliminate the DC offset, which is done by estimating the center of the arc formed by $B_{I}$ and $B_{Q}$, the center of which corresponds to the coordinates ($DC_{I} , DC_{Q}$). For center estimation, the Levenberg–Marquardt (LM) algorithm is used to fit the measured data to Eq. (4)^16^.

4 $$\left( {B_{I} - DC_{I} } \right)^{2} + \left( {B_{Q} - DC_{Q} } \right)^{2} = \left( {A_{R} } \right)^{2}$$

In the fourth stage, the phase signal given by Eq. (5) is obtained, which is based on the DACM algorithm^17^. This algorithm solves the phase unwrapping problem caused by the undefinition of the arctan function when *B*_*I*_* – DC*_*I*_ = *0*.

5 $${\Phi }\left[ n \right] = \mathop \sum \limits_{k = 2}^{n} \frac{{B_{I} \left[ k \right]\left\{ {B_{Q} \left[ k \right] - B_{Q} \left[ {k - 1} \right]} \right\} - \left\{ {B_{I} \left[ k \right] - B_{I} \left[ {k - 1} \right]} \right\}B_{Q} \left[ k \right]}}{{\left( {B_{I} \left[ k \right]} \right)^{2} + \left( {B_{Q} \left[ k \right]} \right)^{2} }}$$

In the fifth stage, the EMD is applied to the phase signal given in Eq. (5) to obtain the intrinsic modes functions of the phase signal. In this work the EMD is performed by using the algorithm proposed in (ref^18^) which is an iterative and adaptive method which was created for the analysis of nonlinear and non-stationary processes. The EMD operates under the hypothesis that any signal is composed of simple intrinsic modes of oscillation. The goal of EMD is to breakdown the original signal into its intrinsic modes functions (IMFs) through a process called sifting. Once the EMD is applied to a signal, it can be expressed as the sum of all the modes generated by the EMD and the residue r^18^. It is important to mention that the EMD automatically removes the DC component since one of the features of the IMFs is that they must have zero mean.

The sixth stage calculates the maximum amplitude A_max_ with Eq. (6) where IMF1 is the first mode of EMD which contains the oscillations caused by RBM. The experiment found that when there are disturbances in the radar signal, they are manifested in IMF1.

6 $$A_{max} = max\left( {IMF1} \right) - min\left( {IMF1} \right)$$

Stage 7 alerts of possible RBM signals when $A_{max} > v_{u}$, where v_u_ is the threshold value defined as the maximum amplitude of IMF1 in the absence of RBM. This value is set by conducting several experiments with PUT at rest.

### Methodology for the RBM elimination and their effects on obtaining breathing rate

As mentioned before, RBM produces spurious signals at high- and low-frequencies; low-frequency ones are the cause of the undesired effect known as CC drift in the phase signal. The first stage of the proposed methodology for RBM elimination is based on the IMFs obtained in the EMD. The proposed methodology defines the spurious-free signal (IM_sf_) as the sum of the second mode (IMF2) and the third mode (IMF3) of the EMD. The spurious-free signal does not consider the first mode or modes greater than 3 since the goal is to eliminate the CC drift and high frequency components that are contained in the first mode.

In the second stage, the signal IM_sff_ is obtained by filtering IM_sf_ by means of a 20-order low pass filter and 1 Hz cutoff frequency to eliminate components outside the breathing range.

Finally, the IM_sff_ spectrogram is obtained by using Short-time Fourier transform (STFT) to estimate the breathing rate of PUT at all times.

## Results

In this section, we show the results obtained by implementing the methodologies proposed in the previous section where *B*_*I*_ and *B*_*Q*_ signals are obtained with the radar operating at 4 GHz.

### Experimental results of the methodology to obtain the person’s breathing rate from signals affected by RBM

In the first stage, the capturing of *B*_*I*_ and *B*_*Q*_ signals were performed, subsequently, the low pass filter was implemented to eliminate high-frequency noise of *B*_*I*_ and *B*_*Q*_. In the third stage, the DC offset was removed and the DACM algorithm was subsequently implemented to perform the demodulation process. Finally, the EMD, code available in (ref^19^), was applied to the phase signal.

**Figure 3:**
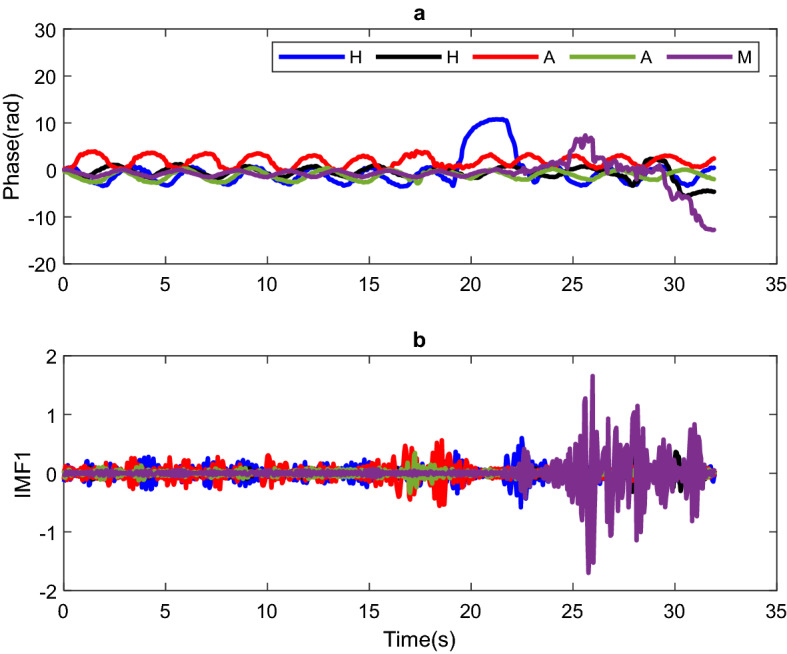
(**a**) Phase signal and (**b**) IMF1 obtained from applying EMD with PUT performing head (H), arm (A) and arms, legs and torso (M) movements.

Once the EMD method was applied to the signal phase and following the sixth stage of the proposed methodology, the RBM detection process was carried out. For this, the $A_{max}$ of the IMF1 is calculated and compared to $v_{u} = 0.7$ to decide whether movement exists. Figure 3a shows the phase signal for two cases where there is a head (H) movement, two cases where there is an arm (A) movement, and one case in which a movement combination of arms, legs, and torso (M) was carried out. Figure 3b shows the IMF1 of the EMD for each of the movements performed by the PUT. Figure 3b shows that IMF1 contains enough information to detect any RBM made by the PUT. It is also important to mention that $v_{u} = 0.7$ value was obtained by 30 measurements with the PUT at rest; 0.7 being the maximum value obtained from the measurements.

### Experimental results of both measure breathing rates methodology and CC-drift/RBM elimination methodology

**Figure 4:**
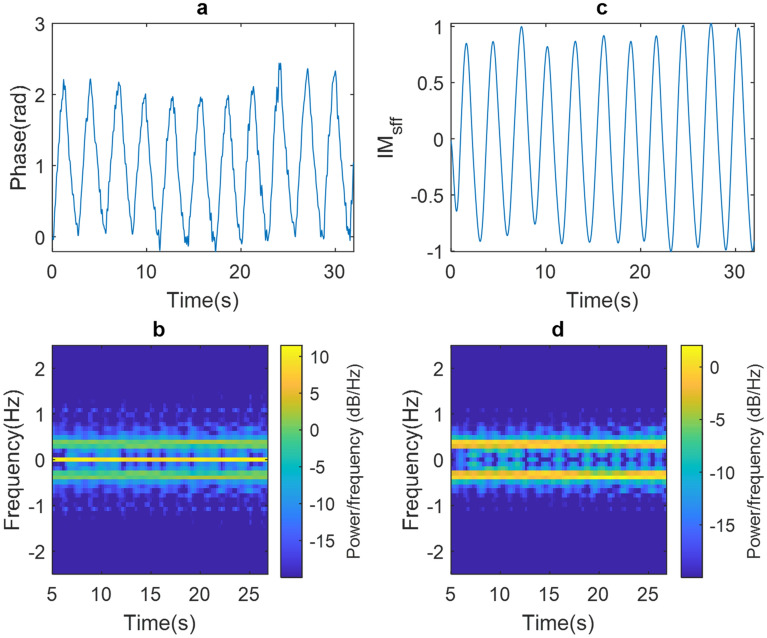
Experimental results of our proposed methodology applied to the PUT at rest: (**a**) raw phase signal, (**b**) raw phase signal spectrogram, (**c**) corrected phase signal and (**d**) corrected phase signal spectrogram.

This methodology uses the IMFs obtained with the EMD. Figure 4a and b show the raw experimental results of the phase signal and spectrogram obtained from the person under test (PUT) at rest. In Fig. 4a the presence of CC can be observed, which is most noticeable in the spectrogram plotted in Fig. 4b. As it can be seen, there is a concentration of power around the frequency 0 Hz at any moment of time. Figure 4c and d show the experimental results of the phase signal and spectrogram corrected with the proposed methodology. Figure 4c shows that the corrected signal does not have a CC, confirmed by its spectrogram shown in Fig. 4d. In this Figure, the components around the frequency 0 Hz have low power while there is an important power spectral density (PSD) magnitude around 0.35 Hz, corresponding to 21 breaths/min.

**Figure 5:**
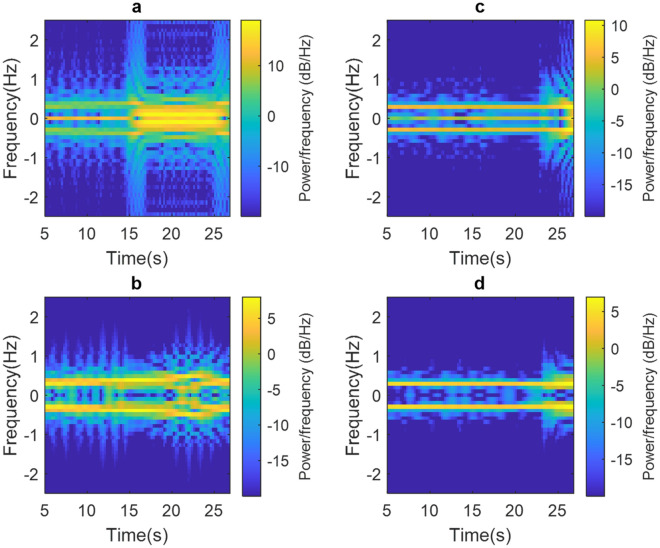
(**a**) Non-corrected spectrogram and (**b**) corrected spectrogram for head movement at 15 s and (**c**) uncorrected spectrogram and (**d**) correction spectrogram for head movement at 25 s.

Figure 5 shows the results obtained after two experiments in which the PUT moves the head perpendicular to its chest. Figure 5a shows that before correction, there is a significant PSD magnitude around the frequency 0 Hz, and at 15 s, the head movement carried out adds CC drift to the phase signal; this can be observed in the spectrogram as an increase in the low-frequency PSD. It is important to mention that in the same spectrogram can be seen the generation of spurious at high frequencies, which are due to the random nature of head movement. Following the application of the proposed methodology, the spectrogram in Fig. 5b shows the mitigation of spurious signals (CC, CC drift and high frequency signals) and that the breathing rate is around 0.35 Hz, which corresponds to 20.82 breaths/min.

On the other hand, Fig. 5c and d show the spectrogram before and after correction, which are the result of the second head movement experiment. The PSD around the frequency of 0 Hz can be observed in the spectrogram in Fig. 5c. In addition, approximately after 25 s, there is a head movement, which adds CC drift and spurious of considerable power. Applying the proposed methodology, we obtain the spectrogram in Fig. 5d, which clearly shows the mitigation of spurious signals and a breathing rate of around 0.30 Hz which corresponds to 18.19 breaths/min.

**Figure 6:**
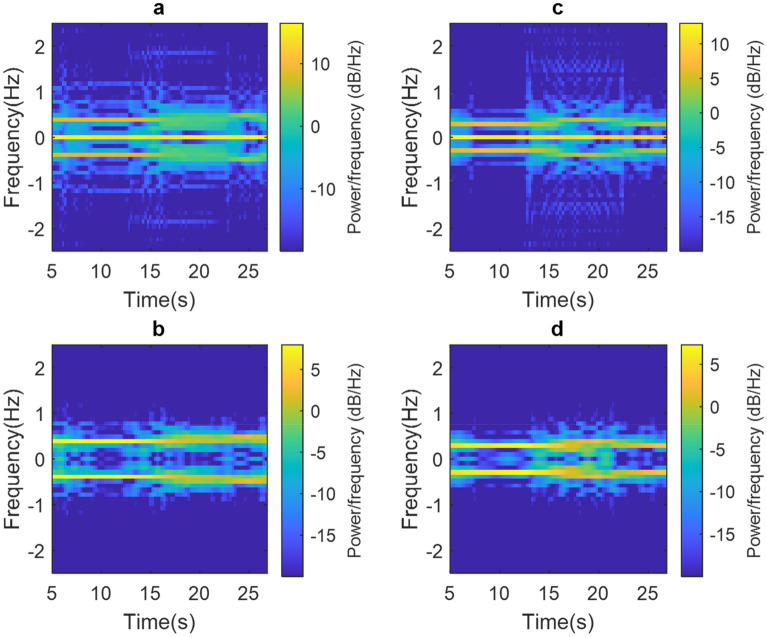
(**a**) Non-corrected spectrogram and (**b**) corrected spectrogram for the first arm movement and (**c**) uncorrected spectrogram and (**d**) correction spectrogram for the second arm movement.

Figure 6 shows the results of two experiments when PUT performs movement in the left arm until it is perpendicular to its chest. Figure 6a shows the spectrogram of the raw phase signal where it is always observed that there is a concentration of power around the frequency 0 Hz; In addition, approximately, when time is equal to 15 s, there is arm movement, which generates CC drift and spurious at high frequencies. On the other hand, Fig. 6b shows that the proposed methodology significantly mitigates spurious signals and that the breathing rate is around 0.39 Hz which corresponds to 23.44 breaths/min.

Similarly, Fig. 6c shows the spectrogram of the raw phase signal for the second experiment. As in the other cases, before correction, there is a high concentration of power at the frequency of 0 Hz, in addition, around 14 s there is CC drift and high frequency spurious caused by the movement of the arm. By applying the proposed methodology, the spectrogram in Fig. 6d was obtained, which shows the mitigation of spurious signals and a breathing rate of 0.29 Hz which corresponds to 17.62 breaths/min.

**Figure 7:**
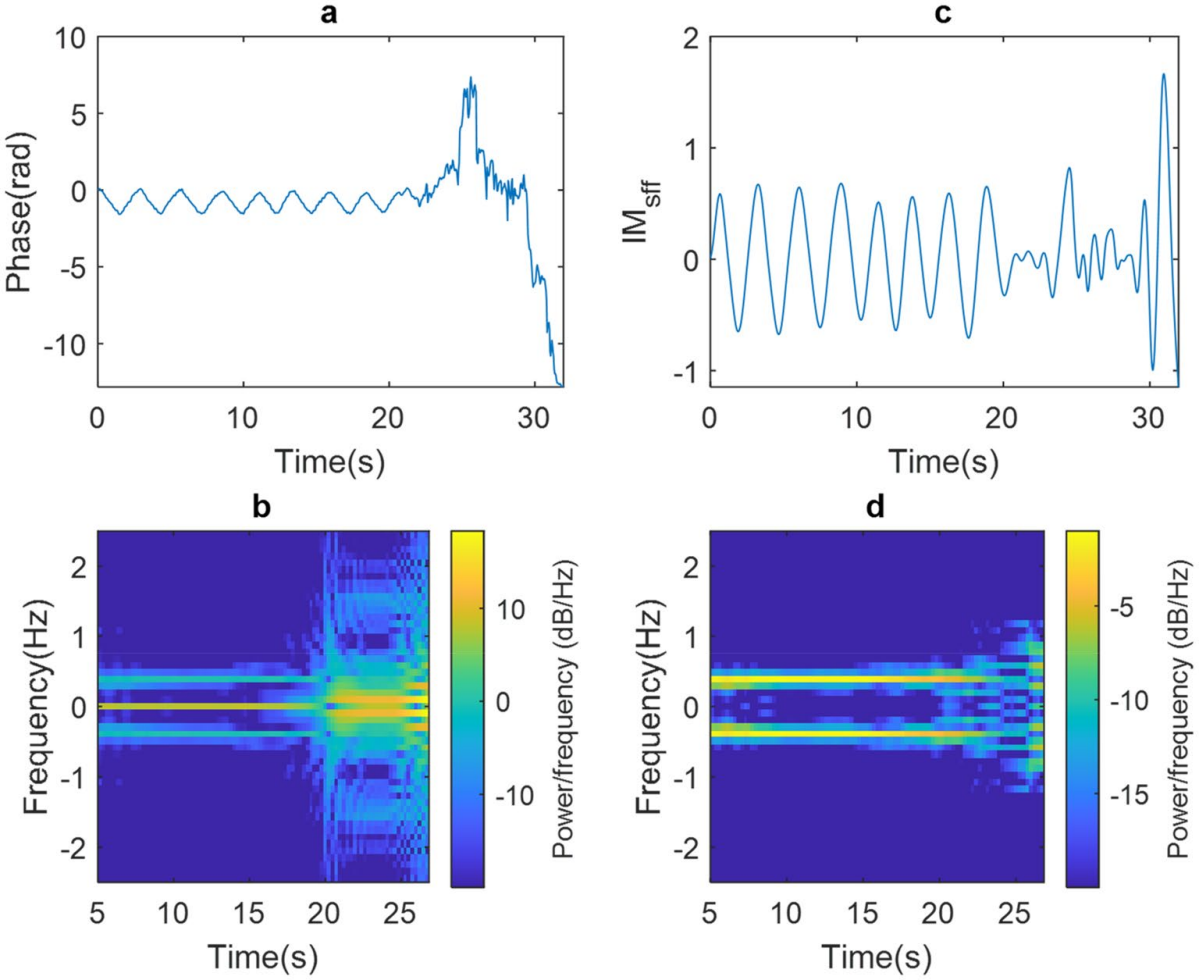
(**a**) raw phase signal, (**b**) raw phase signal spectrogram, (**c**) IM_sff_ and (**d**) IM_sff_ spectrogram of the combined movements experiment.

Finally, Fig. 7 shows the results of an experiment where PUT performs combined movements of arms, legs, and torso. Figure 7a shows the raw phase signal where movements are observed at 22 s. The spectrogram in Fig. 7b shows a high concentration of power at frequency 0 Hz, as well as the appearance of CC drift and spurious at high frequencies. On the other hand, Fig. 7c shows the IM_sff_ signal where the proposed methodology eliminates CC, CC drift and spurious at high frequencies; the above is shown in the spectrogram in Fig. 7d, which made it possible to measure a breathing rate of 0.39 Hz which corresponds to 23.63 breaths/min.

Finally, when comparing our work with the work presented in (ref^9^) we find that both works are similar in the sense that both use EMD. However, there are significant differences. In (ref^9^) the authors are focused on the elimination of small random movements due to artifacts (the radar antenna) while in our work the proposed methodology is focused on the elimination of large random movements (head, arm, and torso). Another big difference is that in (ref^9^) the heart rate is calculated while in the present work the respiration rate is obtained. It is important to mention that the problem of DC drift is omitted in (ref^9^) because the antenna movements are so small that they do not add a significant DC drift. However, in our work the problem of DC drift must be considered because the movements produce significant DC drift in the radar outputs, which in turn causes the appearance of continuous component (CC) drift in the phase signal. In this work the problem of the CC drift is solved through the calculation of the first modes thrown by the EMD since being the CC drift a low frequency signal will appear in higher order modes than those calculated. Additionally, the proposed methodology in (ref^9^) and the proposed in this work are different because the modes of interest are not the same due to the nature of the signals and the nature of the movements.

## Conclusion

A new methodology for the determination of the respiration rate of a PUT, the detection of movements, as well as the elimination of the spurious effects produced by the movements of the PUT was proposed. The methodology is based on EMD applied to the phase signal obtained by means of a quadrature Doppler radar operating in S band. The EMD allows to automatically eliminate the CC which is present in the phase signal since one of the main characteristics of the modes generated by the EMD is that its mean is equal to zero. The first mode of the EMD is used for the detection of movements while the sum of the second and third modes are used for the elimination of the CC drift and the high frequency components produced by the movements of the PUT. The proposed methodology was successfully tested in a PUT at rest and performing movements of the head, arm and combination of head, arm, and torso.
